# Supplementary material for: Genome-Based Exploration of Rhodococcus Species for Plastic-Degrading Genetic Determinants Using Bioinformatic Analysis
Source: Microorganisms. 2022 Sep 15;10(9):1846. doi: 10.3390/microorganisms10091846 (PMC9506104; doi:10.3390/microorganisms10091846)
Supplement: Supplementary file 1 [file Fig S3_Albero_cyt P450_HIT unique_completo.pdf › Fig S3_Albero_cyt P450_HIT unique_completo.pdf]

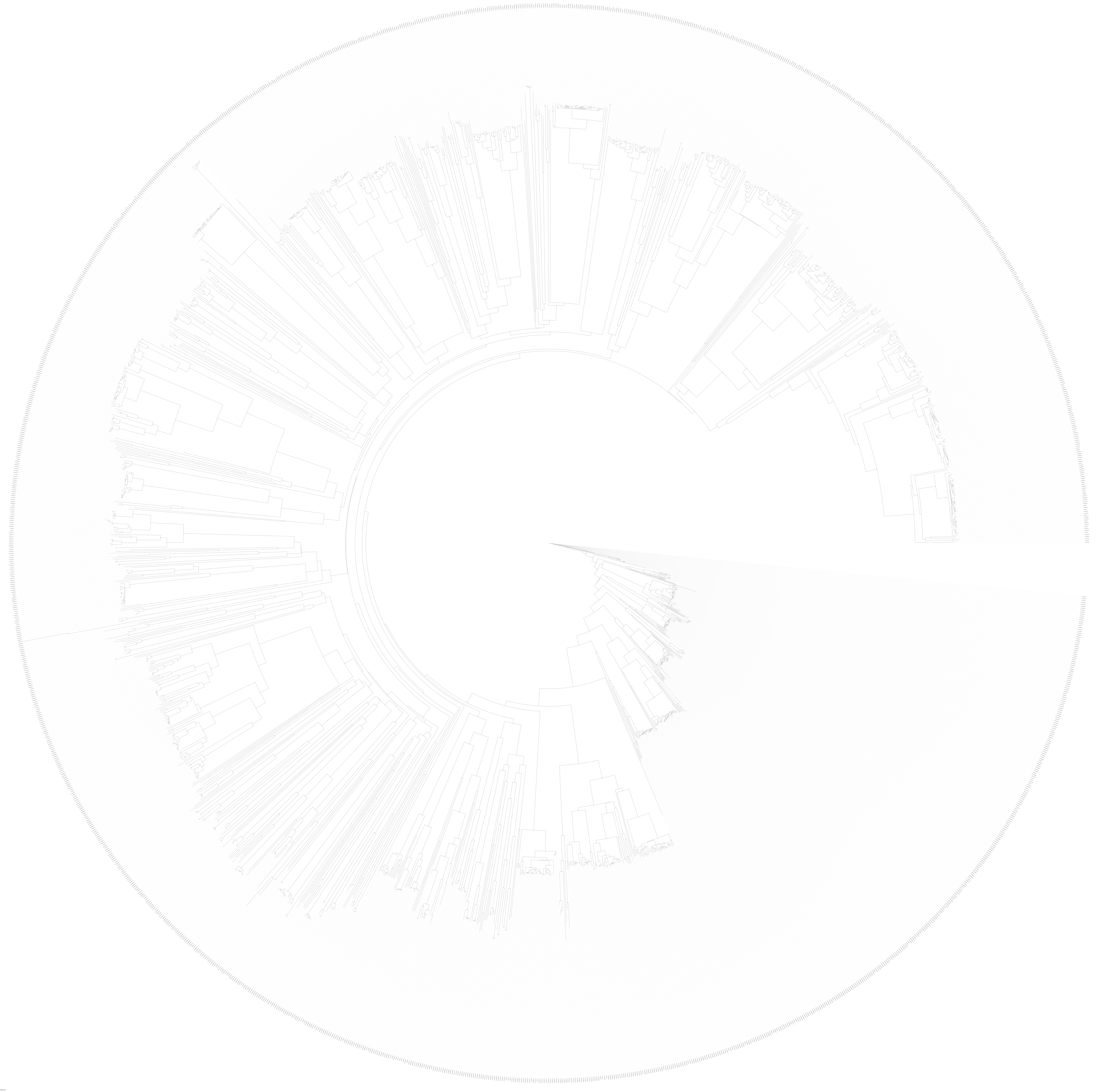
0.008
